# A qualitative study on mothers’ experiences attending an online infant massage class: “It is funny! I feel close to my baby!”

**DOI:** 10.1186/s12912-022-00952-9

**Published:** 2022-07-04

**Authors:** Siti Khuzaiyah, Qorinah Estiningtyas Sakilah Adnani, Nur Chabibah, Milatun Khanifah, Ka Yiu Lee

**Affiliations:** 1 Midwifery Department, Faculty of Health Science, 1st Building of Faculty of Heath Science, Universitas Muhammadiyah Pekajangan Pekalongan, Pekajangan Street No 87, Kedungwuni Pekalongan, Indonesia; 2 Department of Public Health, Faculty of Medicine, Universitas Padjadjaran, Bandung, Indonesia; 3 Swedish Winter Sports Research Centre, Department of Health Sciences, Mid Sweden University, Östersund, Sweden

**Keywords:** Mothers, Infant, Massage, Fear, Anxiety disorders, Anxiety, Depression, COVID-19

## Abstract

**Background:**

The coronavirus disease 2019 (COVID-19) pandemic impacts maternal and perinatal health. Fear of COVID-19 transmission may lead to psychological disorders among mothers, such as anxiety and depression, which might affect the infant's health. Innovation is needed to address problems related to this condition. This study aimed to explore the experiences of mothers who had attended online infant massage classes.

**Methods:**

This qualitative survey recruited 12 Indonesian mothers who had infants aged < 12 months and joined the online infant massage class. An open-ended question form was used to collect data, which were analyzed using thematic content analysis.

**Results:**

There were six themes related to the experiences of mothers attending online infant massage classes: favorite session, new knowledge and skills, benefits, barriers during infant massage class, factors related to infant massage practice, and mother’s hope.

**Conclusions:**

Mothers had a good experience learning infant massage and had better interaction with their infants after the class. The findings show that an online infant massage class could benefit both mothers and infants.

## Background

The coronavirus disease 2019 (COVID-19) pandemic has been a global health concern for mothers and babies. Mothers might have experienced stress, anxiety, depression, and specific behavioral changes during the COVID-19 pandemic [1]. Mother who were struggling with breastfeeding and obtained insufficient support from family or professionals may have worse mental health outcomes [2]. A study on 40 mother-infant dyads in three conditions (during pregnancy, six months, and 14 months of the infant’s life) showed that mothers with chronic depression tend to be less sensitive. They also disengage and have less positive influence on infants than those less affected in the control group. Furthermore, mothers with depression had less sensitivity, less playing with their children, and less positive influence than mothers without mental health problems [3]. Mothers with depression might affect a child’s health, including lower cognitive performance, interaction and creativity, having anxiety disorders and depression, lower IQ score, having phobias, panic disorder, alcohol dependence and learning disorders [4].

Moreover, postpartum stress has a negative association with poor developmental trajectories and linear growth deficits, affecting stunting, poor language, and cognitive development, poor gross and fine motor movement, and infant sleep [5]. Maternal depression also increases the risk of children getting cognitive and language difficulties [6]. Mental health-related problems among mothers should be addressed to prevent further impact and complications such as suicide [7, 8], particularly during the COVID-19 pandemic.

While discussing maternal mental health, an infant's health is also an important issue during the pandemic. When performing infant massage, the mother could interact with their infant intensively. They also make eye contact, skin contact, and meaningful communication through verbal and nonverbal communication. Skin contact can improve the mother-infant attachment and maintain emotional attachment between them [9]. Infant massage might benefit maternal-infant relationships since it combines psychotherapy and education [10]. Some studies indicate that massage improves infants' development, decreases stress behavior, positively impacts the immune system and pain tolerance, and accelerates discharge from the hospital [11]. A randomized placebo-controlled trial among 120 preterm infants shows that natural killer cell cytotoxicity was higher in the massage group, particularly among those who received ≥ 5 consecutive days of intervention, than in the control group. Moreover, the weight of infants in the massage group was heavier than those in the control group after the intervention [12]. Infant massage may help relieve colic and stress in the abdomen, increase dopamine levels, and decrease stress hormone level [13] mainly when a mother performs the massage. Daily infant massage from parents can improve bonding between infants and parents, and the parents are more likely to focus on caring for their babies [13]. Infant massage performed by mothers could decrease the depression level of the mother and provide better interaction [14]. This intervention improves the mother–infant relationship and mood state of mothers [15]. Infant massage by mothers therefore significantly reduced the risk of maternal postpartum depression (PPD) [16].

The International Association of Infant Massage (IAIM) trains and certifies infant massage instructors who deliver infant massage classes to equip parents with infant care-related knowledge and massage skills. During the COVID-19 pandemic, the IAIM regulated that infant massage classes should be conducted online [17]. However, there is no study evaluating these infant massage classes to the best of our knowledge. Therefore, this study aimed to explore mothers' experiences attending online infant massage classes and improve the quality of infant massage classes in the future.

## Methods

### Research design

This was a qualitative study exploring the experiences of 12 mothers attending an online infant massage class during the COVID-19 pandemic in Indonesia. The ethical clearance of this study was issued by the Ethics Committee of the University of Muhammadiyah Semarang (no 238/KEPK-FKM/UNIMUS/2019), and informed consent was obtained from all participants before conducting this study. The online infant massage class was held using Zoom or WhatsApp media. Each participant joined four sessions of infant massage class. The period for data collection was between October 2020 and January 2021. To ensure quality of the research, this study followed 32-items of the consolidated criteria for reporting qualitative research checklist [18].

### Participants and recruitment

The participants of this study were mothers who had infants aged < 12 months and had completed four sessions of infant massage class. Using convenience sampling, participants were recruited based on their availability [19]. Firstly, the researcher reached the participants by publishing the information about the infant massage class through WhatsApp group and social media. The researchers provided a phone number so mothers could make contact if they intended to participate in this research. A brief description of the study and guidelines of the online infant massage class were given. If mothers agreed to participate in this study, the researcher asked them to sign informed consent virtually using a Google Form.

The online infant massage class was delivered by a midwife who has already been certified as a CIMI (Certified Infant Massage Instructor) by the International Association of Infant Massage. The research team organized the classes from the Midwifery Department, University of Muhammadiyah Pekajangan Pekalongan, Central Java, Indonesia. There were four sessions for every class (60 min per session). The duration of the infant massage class was one month (one session per week). During the infant massage class, participants could see each other, both instructor and other participants. The contents of this class were: the concept of infant massage, benefits of infant massage, infant cues, oil utilization for infant massage, contraindication of infant massage, mother-infant bonding, hand containment, colic abdomen, massage preparation, and infant massage stroke. During the class, the instructor gave a chance for each mother to practice infant massage stroke, followed by an evaluation of their performance. The participants were asked to express their feelings and experience related to infant massage classes and activities they performed at home during the period of infant massage class.

The inclusion criteria of this study were as follows: voluntary participation, mothers with babies aged < 12 months, mothers attending all four sessions of infant massage class, and ability to complete an online questionnaire and receive a call. To ensure participants included in this study had completed four sessions of infant massage class, we checked the online attendance list that we had prepared in advance.

### Data collection and analysis

We used a qualitative survey asking open-ended questions to allow space for longer narratives and information from respondent [20]. The list of open-ended questions are presented in Table 1. Utilizing open-ended questions enables researchers to collect holistic and comprehensive information on the studied issues [21]. We used a qualitative survey because this method allows the researcher to collect data from more respondents than qualitative interview studies [20]. The survey was delivered using an online platform (Google Form). This method was chosen because the survey can be completed by individuals anywhere anytime [22]. Moreover, the online survey was more rational because people were restricted from conducting direct meetings in 2020 due to COVID-19.

**Table 1:** Open-ended questions in the qualitative survey

• Tell me which part you liked the most about the infant massage class?
• What have you learned about baby massage in this class?
• Tell us what benefits you get from the infant massage class?
• How do you feel after taking an infant massage class?
• Tell me about your feeling when you massage your baby
• Tell me about the obstacles that mothers experienced during infant massage classes
• Explain what benefits you get by massaging your infant regularly at home?
• Tell me what factors make it easier or more difficult for mothers to massage their children at home?
• Tell me your hopes for your baby in the future

### Data collection and analysis

This study used Colaizzi’s method as it was successfully used in previous research [23]. The steps included: reading and re-reading each text to gain an in-depth sense of the ‘whole’ respondent experiences; putting statements and phrases which directly pertained to the phenomena in a significant statement; reviewing significant statements; identifying emerging themes; sending back the findings to participants for validation.

## Results

### Demographic characteristics

We have reached 30 mothers to participate in this study. However, not all mothers could attend online infant massage classes for all sessions (4 sessions per month). Moreover, not all mothers filled out the survey completely. Finally, only 12 mothers met the inclusion criteria and participated in this study. The participants were aged 24–37 years, and they were primiparous and multiparous. Their infants were aged 1–11 months. All participants were married. Two of them worked in the formal sector, and the rest were unemployed. Four participants delivered their infants by cesarean section, while the others delivered their babies by vaginal birth. One of the infants was delivered prematurely (< 1500 g), while the rest were mature (birth weight, 2840–4000 g). Half of the infants were female. Detailed information about participant characteristics is presented in Table 2.

**Table 2:** The demographic profile of participants (*N* = 12)

Characteristic	n (%)
Age	
20–35	9 (75%)
> 35	3 (25%)
Educational status	
Senior high school	2 (17%)
Diploma	8 (67%)
Bachelor	1 (0.8%)
Master	1 (0.8)
Parity	
Para 1	5 (41.7%)
Para 2	4 (33%)
Para 3	1 (0.8%)
More than 3	2 (17%)
Babies Age	
1 – 6 months	7 (58.3%)
7–12 months	5 (41.7%)
Marital status	
Married	12 (100%)
Occupation	
Not employed	10 (83.4%)
Private sector	1 (0.8%)
Public sector	1 (0.8%)
Type of birth	
Spontaneous delivery	8 (67%)
Section cesarean	4 (33%)
Gestational age when delivering the babies	
Pre-mature	1 (0.8%)
Mature	10 (83.4%)
Post-mature	1(0.8%)
Babies’ type of sexuality	
Male	6 (50%)
Female	6 (50)

### Thematic content analysis

Six themes related to the experiences of mothers attending online infant massage class were identified as follow: favorite session, new knowledge and skills, benefits, barriers during infant massage class, factors related to infant massage practice, and mother's hope (Table 3).

**Table 3:** Themes and subthemes identified

Themes	Subthemes
1.Favorite session	Practicing infant massage Massage technique
2. New knowledge and skills they gathered	Massage technique Information about infant massage Bonding and caring for babies
3. Benefits	Benefits of infant massage class (Understanding infant massage, obtaining new knowledge, excited to perform infant massage) Emotional benefits of infant massage (happy, grateful) Benefits of practicing infant massage (less fussy infant, comfortable, calm, happy)
4. Barriers during infant massage class	Poor Internet connection Infant availability to take part in the class
5. Factors related to infant massage practice	Facilitating factors: The baby has a good mood and free time Hindering factors: The negative mood of babies forgot the strokes and annoying siblings
6. Mother’s hope	Babies grow and develop optimally Babies become active children

### Favorite session

Eight participants stated that they loved the practical session as described below:

“I liked when we practice massage stroke from the beginning until the last” (S, 28 years).

“I enjoyed massage technique as each stroke has each benefit for my baby” (N, 27 years).

“I enjoyed the practice of baby massage because I can apply the theory I gathered from class” (M, 37 years).

Furthermore, two participants enjoyed practicing baby massage in particular areas of the body:

“I liked to massage my baby’s foot” (N, 34 years); “I enjoyed massage baby’s face” (S, 27 years).

### New knowledge and skills

Infant massage instructors provided knowledge and skills related to infant massage. However, when the mothers were asked about their experiences, they provided different responses regarding their knowledge and skills. Mothers reported that they obtained new knowledge about the difference between traditional and modern massage. They also learned how to care for their infants, massage correctly, and the correct time to massage. Furthermore, mothers learned the infant’s development, massage from foot to head, history, benefits, technique, bonding, oil use, and communication with infants.

“I learned many things in this class. For example, I learned how to care for my baby and bounding.” (N, 27 years).

“I learned many things such as the technique of baby massage and the right time to massage.” (S, 27 years).

## Benefits

### Benefits of infant massage class

Infant massage class is beneficial for both infants and mothers. Four participants stated that they understood infant massage very well and could practice it independently.

“Now, I understand about baby massage. I could practice baby massage independently without depending on dukun bayi (a term that refers to a traditional midwife in Indonesia). Moreover, my child became healthier and more active.” (S, 37 years).

One of them reported that she was excited to perform infant massage at home and became calmer when performing infant massage:

“I became calmer and more excited when performing infant massage.” (N, 34 years).

“I gathered new knowledge, and it is beneficial for me as a parent.” (N, 27 years).

Moreover, a mother also reported that the infant massage class was beneficial because she could share her baby-care-related problems with their peers:

“I obtained many benefits from this class. I obtained new knowledge about baby massage, and I can share the daily problem I faced with others.” (N, 25 years).

Infant massage class is also beneficial for mothers to make a good relationship with their peers attending infant massage class. One of the mothers reported the following:

“There were lots of benefits I got from infant massage class. I got new friends, knowledge and I could practice baby massage.” (N, 25 years).

As the curriculum of infant massage included discussion sessions about child development and child care, one of the participants reported that they learned about these elements:

“I got information about infant massage practice, child development, breastfeeding, and communication.” (S, 37 years).

### Emotional benefits of infant massage class

All respondents reported the emotional benefit of the infant massage class. They were happy to join the infant massage class. The mother's expressions were as follows:

“I am happy! Now I become more independent in caring for my baby.” (S, 28 years).

“I am happy because I could practice to my baby directly.” (N, 27 years).

“I am happily joining this class. The tutor delivered the material very well, and she demonstrated infant massage clearly.” (N, 25 years).

“Very happy! It expanded my knowledge and triggered me to learn more and become a kind mother.” (M, 37 years).

### Benefits of practicing infant massage

Most respondents stated that practicing infant massage was beneficial for them. Mothers mentioned some benefits of infant massage, including the benefits on the infant’s sleeping habits, activities, feeding, and mother-infant bonding.

“In my experience, babies become less fussy.” (N, 27 years).

“Thank God! The baby is very comfortable when massaged, and the growth is getting better” (M, 29 years).

“Children feel comfortable, calm, and active, and our bonding is getting better!” (N, 25 years).

“After the massage, the baby sleeps calmly, defecate smoothly, motor movements develop faster.” (E, 37 years).

Five respondents reported the benefits related to their emotions. They were happy and enjoyed performing massage at home:

“It’s enjoyable because we can massage the baby at any time in the right way.” (S, 28 years).

“I am happy, especially when I massage the baby’s face. When my baby has a toothache, he calms down immediately after receiving a massage on his face (S, 27 years).

“My baby was happy when I massaged her.” (K, 24 years).

“We have to understand the best mood of our babies when doing baby massage.” (N, 34 years).

“My son has not been able to get a full massage because he is very active, but he enjoys every massage I give.” (R, 28 years).

## Barriers during infant massage class

### Internet connection

Seven respondents stated that the most significant barrier to joining an online infant massage class was an unstable Internet connection.

“The Internet connection was poor. The time was sometimes not effective because it was prior the lunchtime.” (S, 28 years).

“Poor signal was the greatest problem because it was raining so hard.” (N, 27 years).

“Signal problem. Babies were fussy, so there was no one to help look after them. The material was difficult to understand.” (D, 32 years).

### Infant availability to take part in the massage class

The other barrier were infant availability to take part in the infant massage class.

“I could not practice directly on babies because when the massage class started, the child was fussy, asleep, etc., if the baby massage class was performed offline by practicing the baby directly, it seems like would be more fun, huh….” (K, 24 years).

“I was not focused when the baby was fussy and uncomfortable.” (M, 29 years).

“When the child was fussy, it became less focused.” (S, 37 years).

“The time of the baby massage was the same as the baby’s bedtime.” (N, 34 years).

## Factors related to infant massage practice

Factors that helped mothers to perform infant massage easier were as follows: infant’s good mood and free time. Conversely, factors that made it more difficult were the negative mood of infants, forgetting the strokes, and annoying siblings.

“What makes baby massage easier is when the baby can receive it well and is cooperative, while what complicates the practice of massage is when the baby does not want to be massaged or is fussy.” (S, 28 years).

“The thing that makes it easier for me to massage my baby is because I don’t work so 24 h at home and whenever possible to massage the baby as long as the time is right.” (N, 27 years).

“It is difficult to massage when the baby is not in the mood.” (K, 24 years).

“Makes it easier when the baby’s mood is good.” (N,34 years).

“Doing baby massage is easier during free time, and the baby is not fussy. It will be more difficult if the brothers annoy her and I has troublesome.” (S, 37 years).

“It’s funny. I feel very close with my baby. What makes it difficult is that brother interferes.” (R, 28 years).

## Mother’s hope

All respondents hoped that their children grow smoothly as normal children and are always healthy. They mentioned that, by performing infant massage regularly, they would like their children to develop optimally and become active.

“I hope that my baby will be healthier in the future, not easily sick, grow and develop according to the stages of his age.” (S, 28 years).

“Hopefully the development is appropriate and even faster. And.. he will has better growth. Amen.” (N, 27 years).

“I hope he becomes a healthy child, grows and develops well and becomes an active child.” (K, 24 years).

“I hope my son has more appetite, is more active and smart.” (D, 32 years).

“I hope that children can grow and develop optimally, healthy, strong and intelligent.” (R, 28 years).

## Discussion

This study described mothers’ experience after joining online infant massage classes. The main result of this study is that there were six themes related to mothers' experiences attending online infant massage classes: favorite sessions, new knowledge and skills, benefits, barriers during infant massage class, factors related to infant massage practice, and mother's hope. This study is different from previous studies because it explored the online infant massage class, whilst others focused on physical infant massage classes. Generally, the mothers in this study were happy and equipped with knowledge and skills in infant massage. A previous similar study examined the mother’s experience in attending infant massage classes in person [24]. This study adds further insights by investigating an online infant massage class.

Most mothers reported that practicing infant massage is their favorite session. It indicates that practical sessions are essential for mothers to acquire skills and develop bonding with infants. A previous study revealed that mothers enjoyed infant massage classes and felt closer to infants [24]. Mothers can make eye contact with infants when they conduct the massage. During the process, mothers and infants are together and they have intensive skin to skin contact which could establish a bond between a mother and her newborn [25].

Furthermore, skin-to-skin contact, breastfeeding, and infant massage during the first postpartum hour could promote bonding between mothers and newborns [26]. Eventually, this bonding has lifelong implications for both mothers and infants. Bonding with a parent or primary caregiver in the first year would also impact the infant’s different aspects of brain development, including social, emotional, and cognitive development, and lead to happiness, independence, and resilient in adulthood [27].

Mothers stated that they learned new skills and knowledge related to infant massage during the online infant massage class in this study. A previous study indicates an increase in knowledge and skills after attending the infant massage class [28], of which are beneficial for mothers to perform infant massage independently.

Besides acquiring knowledge and skill related to infant massage, mothers also learned about infant’s daily care through peer sharing their own experiences caring for their infants and supporting each other. While the instructor provides education about infant massage, she also has the role of peer support which empowers women to be competent mothers. Peer support, which could be provided online [29], is a flexible concept used in many healthcare fields, including breastfeeding and parenthood [30].

After joining the class, most mothers indicated that they were happy through a discussion of feelings. The findings were consistent with a previous study that showed that performing infant massage is a heart-warming, enjoyable, and satisfying moment [31]. In terms of obstacles in joining online infant massage, the internet connection appears to be the main issue, as all mothers live in rural areas where they have limited internet access. Spontaneous disconnection from the Zoom meeting distracted both the instructor and participants. The availability and slow-speed internet connection have been problematic in online learning [32]. There were also practical issues when conducting the massage class. On some occasions, infants were sleepy and fussy, and the mother could not practice massage.

The limitation of this study pertains to the data collection conducted through an online platform and telephone call in which the body language of respondents could not be captured. Body language delivers a nonverbal message which provides further understanding of the expression of individuals. Despite the limitation of the study, the results of this study could be used as a reference to conduct better online infant massage classes in the future, by preparing a good internet connection and scheduling the right time for babies.

## Conclusion and recommendations

Indonesian mothers in this study had a good experience in learning infant massage on an online platform. Nurses and midwives could consider joining the infant massage training and encourage mothers to perform infant massage, which is affordable, health-promoting, and safe [24].

## Data Availability

The datasets generated and/or analysed during the current study are not publicly available due to confidentiality but are available from the corresponding author on reasonable request.

## Abbreviations

COVID-19: Coronavirus disease 2019
IAIM: The International Association of Infant Massage
